# Rates of acute pancreatitis and cardiovascular events among adults with severe or extreme hypertriglyceridemia in US clinical practice

**DOI:** 10.1186/s12944-025-02658-8

**Published:** 2025-07-28

**Authors:** Asia Sikora Kessler, Seth J. Baum, Emily Kutrieb, Montserrat Vera Llonch, Alex Lonshteyn, Derek Weycker, Daniel E. Soffer

**Affiliations:** 1 Ionis Pharmaceuticals Inc., Boston, MA USA; 2 Flourish Research, Boca Raton, FL USA; 3 Avalere Health, Boston, MA USA; 4 University of Pennsylvania Health System, Philadelphia, PA USA

**Keywords:** Hypertriglyceridemia, Triglycerides, Cardiovascular disease, Acute pancreatitis, Adults

## Abstract

**Background:**

Severe and extreme hypertriglyceridemia (sHTG [TG 500–879 mg/dL; 5.65–9.93 mmol/L]; eHTG [TG ≥ 880 mg/dL; ≥ 9.94 mmol/L]) are important risk factors for acute pancreatitis (AP) and cardiovascular (CV) events. The objective of this study was to estimate rates of AP and CV events for adults with (and without) sHTG/eHTG in US clinical practice.

**Methods:**

A retrospective design and data from the MarketScan Research Databases were employed. Study population comprised adults with ≥ 1 TG value and was stratified by index TG (< 150, 150–499, 500–879, ≥ 880 mg/dL; < 1.69, 1.69–5.64, 5.65–9.93, ≥ 9.94 mmol/L). AP/CV events (per 1,000 person-years [PY]) were ascertained from index TG through end of study period, and were estimated for TG-specific subgroups and selected subsets defined therein.

**Results:**

Study population totaled 1.8 M adults (TG < 150 mg/dL [< 1.69 mmol/L]: *N* = 1.3 M; TG 150–499 mg/dL [1.69–5.64 mmol/L]: *N* = 449 K; TG 500–879 mg/dL [5.65–9.93 mmol/L]: *N* = 12,050; TG ≥ 880 mg/dL [≥ 9.94 mmol/L]: *N* = 3,944). AP rates (per 1,000 PY) increased from lowest to highest TG value (0.6 [< 150 mg/dL; < 1.69 mmol/L]) to 9.9 [≥ 880 mg/dL; ≥ 9.94 mmol/L]); rates were highest for adults with TG ≥ 880 mg/dL (≥ 9.94 mmol/L) and history of AP (193.0), pre-existing diabetes (13.9), or history of LLT (13.9). CV event rates (per 1,000 PY) also increased from lowest to highest TG value (3.3 [< 150 mg/dL; < 1.69 mmol/L]) to 10.3 [≥ 880 mg/dL; ≥ 9.94 mmol/L]); rates were highest for adults with TG ≥ 880 mg/dL (≥ 9.94 mmol/L) and history of CV events (116.5), pre-existing diabetes (18.1), or history of LLT (14.5).

**Conclusion:**

Rates of AP/CV events are substantially higher among adults with elevated TG values, and are especially high among adults with sHTG or eHTG, in particular those with these conditions and other risk factors. Understanding the magnitude of disease risk among sHTG/eHTG patients, with increasing TG levels as well as within important subgroups, is critical to improving patient care and outcomes.

**Supplementary Information:**

The online version contains supplementary material available at 10.1186/s12944-025-02658-8.

## Introduction

Hypertriglyceridemia (HTG), defined as triglyceride (TG) levels ≥ 150 mg/dL (≥ 1.69 mmol/L), is associated with serious clinical manifestations including acute pancreatitis (AP) and cardiovascular disease (CVD) [1, 2]. Roughly one quarter of adults in the United States (US) have elevated TGs, 1–2% have severe hypertriglyceridemia (sHTG; TG 500–879 mg/dL [5.65–9.93 mmol/L]), and 0.3% have extreme hypertriglyceridemia (eHTG; TG ≥ 880 mg/dL [≥ 9.94 mmol/L]) [3–5]. Adults with certain comorbidities (e.g., insulin resistance, diabetes mellitus, obesity) have a much higher risk of HTG, which is particularly concerning given the increasing prevalence of these comorbidities [6–10]. In fact, HTG may be caused by those conditions and/or by an inherited predisposition. Therefore, whether to treat the TG level directly or to address the risks of AP and CV indirectly by treating the secondary cause is a matter that has not been fully established, although recent evidence from randomized clinical trials has shown that TG reduction lowers AP risk in patients with primary HTG (e.g., familial chylomicronemia syndrome) [11, 12].

For patients with HTG, guidelines recommend lifestyle modifications and, if appropriate, use of lipid-lowering therapy (LLT) including statins, fibrates, and/or omega-3 fatty acids, depending on TG level and the relative risk for AP and CVD [1, 2]. However, effectiveness of LLT among patients with HTG is often limited due in large part to low rates of adherence and high levels of discontinuation as well as poor control of contributing metabolic conditions (e.g., insulin resistance, diabetes, obesity) [8, 13, 14]. Because of the high prevalence, potential serious consequences, and limited treatment options, HTG continues to be a public health concern.

Findings from several recent studies provide additional evidence on the risks of AP and CVD among patients with HTG as well as the relative risks of these diseases by TG level [5–8, 15–18]. These studies, however, are limited as they employed older data, included small sample sizes, focused on unique subgroups (e.g., statin-treated patients), and/or did not evaluate risks/relative risks within TG-specific subgroups defined on other risk factors (e.g., diabetes). The current real-world evidence study was therefore undertaken to better understand the magnitude of AP/CV event rates among adults with HTG, sHTG, and eHTG (as well as among those with lower TG levels), on an overall basis and within subgroups defined on other risk factors.

## Methods

### Study design and data sources

This study employed a retrospective observational cohort design and data (2013–2019) from the Merative MarketScan Research Databases—the Commercial Claims and Encounters (CCAE) and Medicare Supplemental and Coordination of Benefits (MDCR) Databases, linked (at the patient level) with the Labs Database. The study period end date (2019) was selected to avoid potential confounding of study results from use of data during the COVID-19 pandemic.

The CCAE Database includes healthcare claims and enrollment information from employer-sponsored commercial plans throughout the US that provide health benefits to working persons aged < 65 years, including the employees as well as their spouses and dependents. The MDCR Database includes healthcare claims and enrollment information for Medicare-eligible retirees who enrolled in an employer-sponsored Medicare supplemental plan (and for which Medicare- and employer-paid amounts are available). The Labs Database includes records from laboratory tests for a subset of patients in the CCAE/MDCR Databases, and for those patients, lab results are available from a subset of providers.

Medical (i.e., facility and professional-service) claims include the dates/places of service, diagnoses, procedures, and quantity of services (professional-service claims). Outpatient pharmacy claims include the drug dispensed, dispensing date, dose, quantity dispensed, and therapy days. Medical and pharmacy claims also include amounts paid by health plans and patients to healthcare providers for services rendered. Lab records include the laboratory test, test result, and test date. Selected demographic and eligibility information is available for all covered lives.

### Study population

The study population comprised adults aged ≥ 18 years who had ≥ 1 TG value between January 1, 2014 and December 31, 2018. The first TG value was designated the “index TG,” and the corresponding date, the “index date.” Patients were excluded from the study population if they had: TG values < 35 mg/dL (< 0.4 mmol/L) or > 15,000 mg/dL (> 169.5 mmol/L), inconsistent sodium concentration values within 30 days of their index date, or < 12 months of continuous healthcare enrollment before their index date. Inconsistent sodium values were defined as concentrations of 135–145 mmol/L in the presence of TG 5,000–10,000 mg/dL (56.5–113 mmol/L), or concentrations ≥ 130 mmol/L in the presence of TG ≥ 10,000 mg/dL (≥ 113 mmol/L). This criterion was employed to exclude patients with potentially erroneous TG values since hyponatremia is associated with chylomicronemia and thus those not exhibiting extremely low sodium may be an indication of an inaccurate TG value.

Qualifying patients were stratified based on their index TG (< 150, 150–499, 500–879, ≥ 880 mg/dL [< 1.69, 1.69–5.64, 5.65–9.93, ≥ 9.94 mmol/L]). Categories for index TGs were based on definitions for HTG (≥ 150 mg/dL [≥ 1.69 mmol/L]) and sHTG (≥ 500 mg/dL [≥ 5.65 mmol/L]) from the American College of Cardiology; the definition for sHTG (500–879 mg/dL [5.65–9.93 mmol/L]) from the European Atherosclerosis Society; as well as the definition for eHTG (≥ 880 mg/dL [≥ 9.94 mmol/L]) from the European Atherosclerosis Society [1, 2]. An alternative stratification scheme that allocates patients with TG ≥ 880 mg/dL (≥ 9.94 mmol/L) into two subgroups—TG 880–1771 mg/dL (9.94–19.99 mmol/L) vs. TG > 1771 mg/dL (> 20 mmol/L)—was also employed.

### Study measures

Study measures were ascertained from index date through the end of follow-up, which was defined as the date of health plan disenrollment or the end of the study period (whichever occurred first) and included AP and CV events. AP was identified based on acute-care hospitalizations with a principal diagnosis code for AP, or a secondary diagnosis code for AP in combination with a principal diagnosis code for an AP-related complication. AP-related complications included acute necrotic colitis, ascites, organ failure, sepsis, and systemic inflammatory response syndrome (SIRS). CV events were identified based on acute-care hospitalizations with a principal diagnosis code for heart disease (e.g., myocardial infarction, unstable angina), cerebrovascular disease (e.g., stroke, transient ischemic attack [TIA]), or heart failure. Operational algorithms and codes for identifying AP and CV events are set forth in the Supplement (Table 1).

### Baseline characteristics

Baseline characteristics were assessed during the 12-month period preceding the index date and included age, sex, comorbidity profile, and use of LLT. Comorbidities (e.g., diabetes) were identified based on ≥ 1 encounter with a corresponding diagnosis code, irrespective of care setting or diagnosis code position (Supplement – Table 2). LLT use was identified based on ≥ 1 filled outpatient prescription with a corresponding National Drug Code.

### Statistical analyses

Baseline characteristics were summarized using means (standard deviations [SD]) and percentages. Rates of AP/CV events were expressed per 1,000 person years (PY), and were estimated for each TG-specific subgroup, overall and for subsets defined therein based on age (< / ≥ 40 years), pre-existing diabetes, history of AP (as defined in Study Measures), history of CV event (as defined in Study Measures), and history of lipid-lowering therapy (LLT), respectively. Rates and relative rates (i.e., incidence rate ratios [IRRs], by TG level) were estimated using Poisson regression without adjustment for other variables.

## Results

### Study population

Among 3.3 M adults with ≥ 1 TG value between January 2014 and December 2018, 1.8 M met all qualifying criteria and were included in the study population; by index TG, the population included 1.3 M (74%) patients with TG < 150 mg/dL (< 1.69 mmol/L), 449 K (26%) with TG 150–499 mg/dL (1.69–5.64 mmol/L), 12,050 (0.7%) with TG 500–879 mg/dL (5.65–9.93 mmol/L), and 3,944 (0.2%) with TG ≥ 880 mg/dL (≥ 9.94 mmol/L) (Table 1). Mean age ranged from 48 years [TG < 150 mg/dL (< 1.69 mmol/L)] to 51 years [TG 150–499 mg/dL (1.69–5.64 mmol/L)], and percentage male ranged from 41% [TG < 150 mg/dL (< 1.69 mmol/L)] to 74% [TG 500–879 mg/dL (5.65–9.93 mmol/L)] (Table 2).

**Table 1:** Selection of study population

Selection Criteria	Number
Study Population	
Patients aged ≥ 18 years with ≥ 1 TG value from 1/1/14 to 12/31/18	3,288,144
Pus all TG values ≥ 35 mg/dL or (≥ 0.4 mmol/L) or ≤ 15,000 mg/dL (≤ 169.5 mmol/L)	3,165,075
Plus no inconsistent sodium concentration values within 30 days of index date^a^	3,165,041
Plus had ≥ 12 months of continuous enrollment before first TG value	1,755,743
Index TG Value	
< 150 mg/dL (< 1.69 mmol/L)	1,290,630
150–499 mg/dL (1.69–5.64 mmol/L)	449,119
500–879 mg/dL (5.65–9.93 mmol/L)	12,050
≥ 880 mg/dL (≥ 9.94 mmol/L)	3,944

*TG* Triglyceride

^a^Sodium concentration of 135–145 mmol/L considered inconsistent with TG value 5,000–10,000 mg/dL; ≥ 130 mmol/L considered inconsistent with TG value ≥ 10,000 mg/dL

**Table 2:** Baseline characteristics of study population

	Index TG Value			
	<150 mg/dL	150-499 mg/dL	500-879 mg/dL	≥880 mg/dL
	(<1.69 mmol/L)	(1.69-5.64 mmol/L)	(5.65-9.93 mmol/L)	(≥9.94 mmol/L)
	(*N*=1,290,630)	(*N*=449,119)	(*N*=12,050)	(*N*=3,944)
**Age (years), mean (SD)**	47.6 (14.4)	50.7 (12.6)	49.3 (10.9)	47.8 (10.5)
**Male, %**	40.9	55.7	73.8	73.2
**Comorbidity Profile, %**				
Alcoholism	0.9	1.0	1.9	2.3
Atherosclerosis	3.7	5.3	5.9	5.3
Cardiovascular disease	10.6	13.3	13.8	12.1
Cerebrovascular disease	2.9	3.6	3.8	2.9
Heart disease (chronic)	9.1	11.5	12.0	10.5
Heart failure	1.3	1.8	1.7	1.9
Gallbladder disease	1.2	1.5	1.4	1.5
Hyperthyroidism	0.7	0.6	0.6	0.3
Hypothyroidism	11.5	12.9	10.8	9.5
Immunosuppressive conditions/treatments	3.8	4.5	3.9	3.2
Liver disease (chronic)	1.8	2.6	3.3	3.7
Malabsorption	0.5	0.3	0.3	0.2
Metabolic disorders	54.7	72.7	79.8	76.5
Diabetes (exclude pregnancy and complication of DM)	12.1	21.4	33.8	37.2
Disorders of lipoprotein metabolism and other lipidemias	33.5	50.2	57.7	55.0
Hypertensive diseases	32.6	46.3	52.1	48.0
Hyperglycemia, unspecified	2.8	3.9	4.7	4.7
Overweight or Obesity	16.0	21.8	22.4	21.1
Neurologic disorders (chronic)	2.3	2.4	2.2	2.0
Osteoarthritis	8.3	9.8	8.5	6.5
Pancreatitis	0.2	0.4	0.9	2.3
Acute pancreatitis	0.2	0.3	0.8	2.2
Chronic pancreatitis	0.1	0.1	0.3	0.7
Renal disease (chronic)	3.2	4.9	6.4	6.1
Respiratory disease	9.7	11.0	10.9	9.6
**Medications During 12-Month Pre-Index Period, %**				
Lipid-lowering therapy, by category	16.2	27.1	32.7	33.1
Statins	15.2	24.1	24.9	24.5
Statins alone	13.8	20.2	16.5	13.4
TG-targeting therapy^a^	1.4	5.2	14.8	18.1
TG-targeting therapy alone^a^	0.5	2.0	6.6	7.3
Other medications				
GLP-1 receptor agonists	0.4	1.0	1.7	1.6
Medications that could increase TGs	11.2	9.9	7.0	7.4
**Laboratory Values (mg/dL)**				
Total cholesterol				
Mean (SD)	184.2 (36.9)	204.1 (41.7)	233.9 (52.0)	293.1 (98.1)
N	1,283,374	446,665	11,992	3,240
HDL				
Mean (SD)	58.4 (16.9)	44.9 (12.3)	34.5 (9.6)	29.1 (8.5)
N	1,274,959	444,095	11,887	3,168
LDL				
Mean (SD)	109.2 (32.8)	120.8 (36.2)	97.3 (36.9)	67.4 (41.8)
N	1,283,788	446,932	11,917	3,153
TG				
Mean (SD)	89.9 (29.0)	219.9 (69.0)	620.7 (98.6)	2,667.7 (3,038.7)
N	1,290,630	449,119	12,050	3,944
**Months of follow-up, mean (SD)**	32.0 (23.0)	32.5 (23.4)	31.4 (23.2)	29.8 (22.1)

*DM *Diabetes mellitus, *SD *Standard deviation, *TG *Trigylceride

^a^Most common TG-targeting therapy was fenofibrate, ranging from 0.8% (<150) to 10.7% (≥880)

Common comorbidities included hypertension (33–52%), diabetes (12–37%), overweight/obesity (16–22%), cardiovascular disease (11–14%), hypothyroidism (10–13%), and respiratory disease (10–11%). Additionally, 0.2% (TG < 150 mg/dL [< 1.69 mmol/L]) to 2.3% (TG ≥ 880 mg/dL [≥ 9.94 mmol/L]) of patients had a history of pancreatitis (acute or chronic). Use of LLT ranged from 16–33%, increasing from the lowest to the highest TG subgroup. Mean duration of follow-up ranged from 30–32 months across TG subgroups.

### Rates of AP

**Fig. 1:**
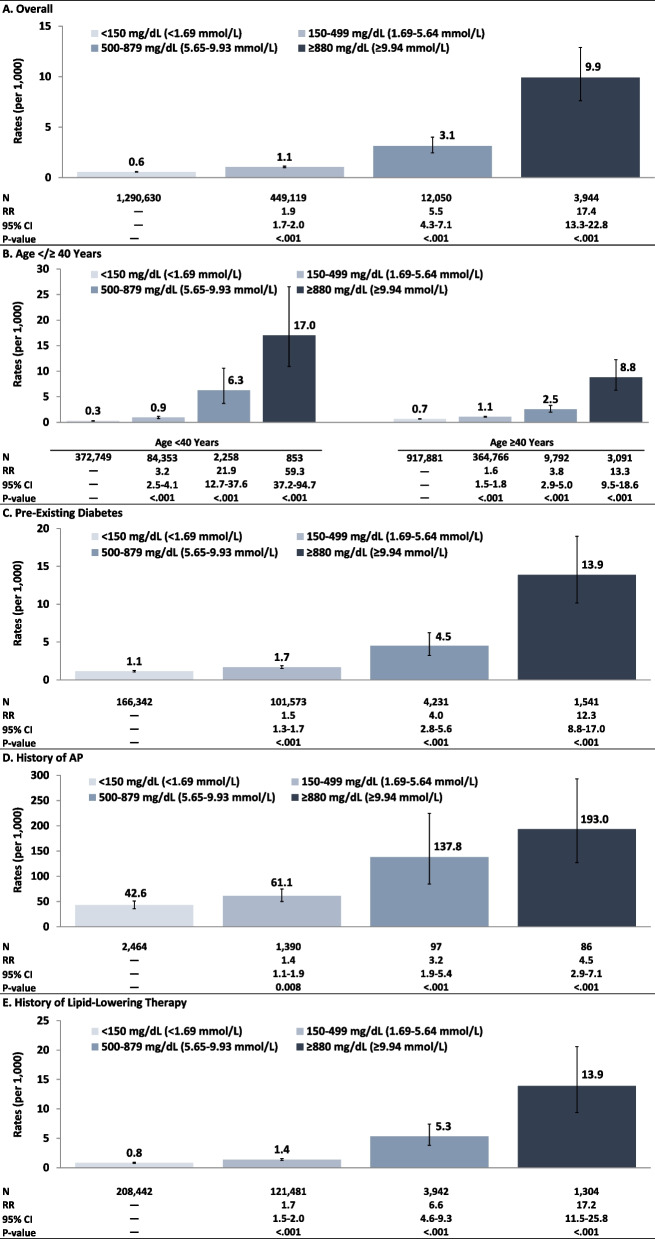
Rates/relative rates of AP by index TG value, overall and within selected subgroups. *Legend:* AP: acute pancreatitis; TG: triglyceride

On an overall basis, rates (95% confidence intervals [CI]) of AP per 1,000 PY increased monotonically from lowest TG value (< 150 mg/dL [< 1.69 mmol/L]: 0.6 [0.5–0.6]) to highest TG value (≥ 880 mg/dL [≥ 9.94 mmol/L]: 9.9 [7.6–12.9]), corresponding to a relative rate of 17.4 (13.3–22.8) for adults with TG ≥ 880 mg/dL versus TG < 150 mg/dL (< 1.69 mmol/L) (Fig. 1, Supplement – Table 3). AP rates also increased from lowest to highest TG value within strata defined on age, diabetes, history of AP, and history of LLT. Relative rates (TG ≥ 880 mg/dL [≥ 9.94 mmol/L] vs. < 150 mg/dL [< 1.69 mmol/L]) ranged from 4.5 (2.9–7.1) among adults with a history of AP to 59.3 (37.2–94.7) among those aged < 40 years.

AP rates were particularly high among adults with a history of AP, irrespective of TG level, ranging from 42.6 (35.8–50.8) for those with TG < 150 mg/dL (< 1.69 mmol/L) to 193.0 (127.3–292.8) for those with TG ≥ 880 mg/dL (≥ 9.94 mmol/L). Rates were lower (vs. those for patients with a history of AP) but still elevated for patients with TG ≥ 880 mg/dL (≥ 9.94 mmol/L) who also had pre-existing diabetes (13.9 [10.2–19.0]) or a history of lipid-lowering therapy (13.9 [9.4–20.6]). When allocating patients with TG ≥ 880 mg/dL (≥ 9.94 mmol/L) between two subgroups, AP rate was 9.1 (6.5–12.6) among patients with TG 880–1771 mg/dL (9.94–19.99 mmol/L) and 12.0 (7.8–18.5) among those with TG > 1771 mg/dL (> 20 mmol/L) (Supplement – Table 4).

### Rates of CV events

**Fig. 2:**
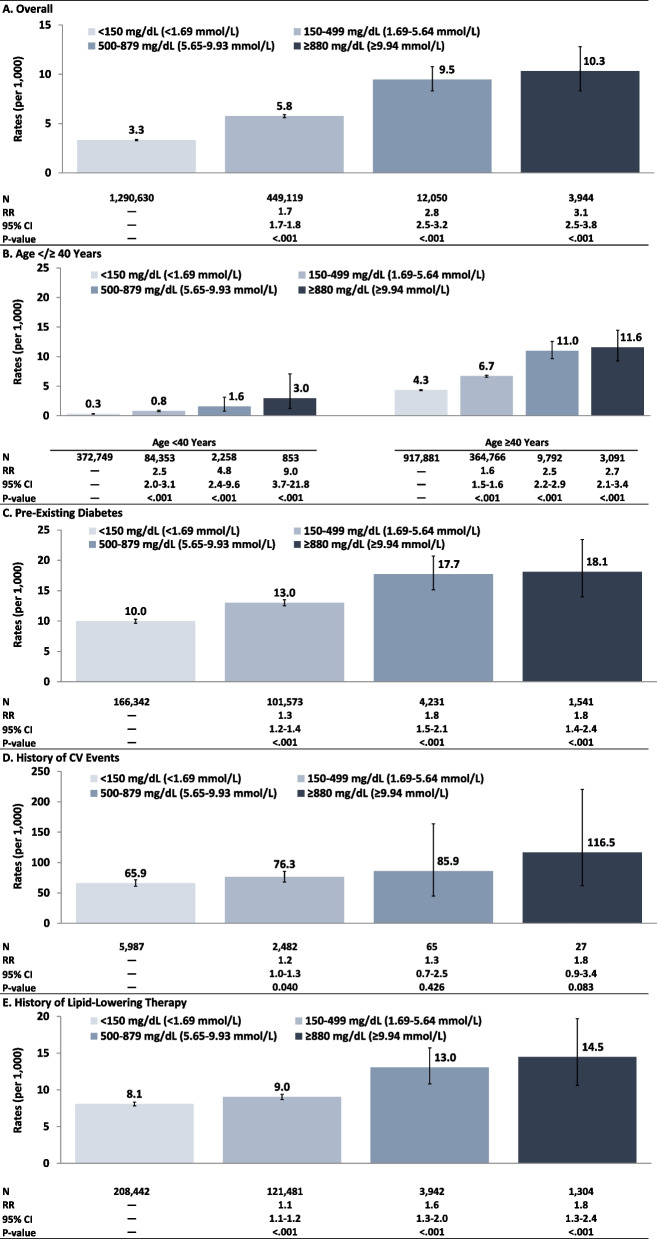
Rates/relative rates of CV events by index TG value, overall and within selected subgroups. *Legend:* CV: cardiovascular event; TG: triglyceride

Rates of CV events per 1,000 PY—on an overall basis—also increased monotonically from lowest TG value (< 150 mg/dL [< 1.69 mmol/L]: 3.3 [3.3–3.4]) to highest TG value (≥ 880 mg/dL [≥ 9.94 mmol/L]: 10.3 [8.3–12.8]), corresponding to a relative rate of 3.1 (2.5–3.8) for adults with TG ≥ 880 mg/dL (≥ 9.94 mmol/L) versus TG < 150 mg/dL (< 1.69 mmol/L) (Fig. 2). Increases in CV rates plateaued, however, when comparing TG 500–879 mg/dL (5.65–9.93 mmol/L) versus TG ≥ 880 mg/dL (≥ 9.94 mmol/L). CV event rates also increased from lowest to highest TG value within strata defined on age, diabetes, history of AP, and history of LLT. Relative rates (TG ≥ 880 mg/dL [≥ 9.94 mmol/L] vs. < 150 mg/dL [< 1.69 mmol/L]) ranged from 1.8 (0.9–3.4) among adults with a history of CV events to 9.0 (3.7–21.8) among those aged < 40 years.

CV rates were particularly high among adults with a history of CV events, irrespective of TG level, ranging from 65.9 (60.8–71.4) for those with TG < 150 mg/dL (< 1.69 mmol/L) to 116.5 (61.6–220.5) for those with TG ≥ 880 mg/dL (≥ 9.94 mmol/L). Rates were lower (vs. those for patients with a history of CV events) but still elevated for patients with TG ≥ 880 mg/dL (≥ 9.94 mmol/L) who also had pre-existing diabetes (18.1 [14.0–23.4]) or a history of lipid-lowering therapy (14.5 [10.6–19.7]). When allocating patients with TG ≥ 880 mg/dL (≥ 9.94 mmol/L) between two subgroups, CV event rate was 10.2 (7.8–13.3) among patients with TG 880–1771 mg/dL (9.94–19.99 mmol/L) and 10.6 (7.4–15.2) among those with TG > 1771 mg/dL (> 20 mmol/L).

## Discussion

The current study was undertaken to better understand rates and relative rates of AP and CV events among adults with elevated TG levels in US clinical practice, on an overall basis and among important subgroups. Study findings indicate that rates of AP increase monotonically with increasing TG levels, and that rates are particularly high among adults with sHTG and eHTG, especially when in the presence of other risk factors or proxies for risk factors (e.g., history of AP). Similarly, with few exceptions, monotonic increases in rates and relative rates of CV events were observed with higher TG levels although increases plateaued when comparing TG 500–879 mg/dL (5.65–9.93 mmol/L) versus TG ≥ 880 mg/dL (≥ 9.94 mmol/L). Exceptions included patients with a history of CV events and adults aged < 40 years, in whom CV event rates continued to increase with TG ≥ 880 mg/dL (≥ 9.94 mmol/L).

Notwithstanding differences in study designs and methods, the relationship between TG levels and AP reported herein is comparable to findings reported in previous research. In a recent retrospective study by Sanchez et al., which employed comparable definitions for TG subgroups, crude incidence rates for AP per 1,000 person-years were 0.7 for TG < 200 mg/dL (< 2.26 mmol/L) (vs. 0.6 for TG < 150 mg/dL [< 1.69 mmol/L] in the present study), 1.1 for TG 200–500 mg/dL (2.26–5.65) (vs. 1.1 for TG 150–499 mg/dL [1.69–5.64 mmol/L]), 2.3 for TG > 500–880 mg/dL (> 5.56–9.94 mmol/L) (vs. 3.1 for TG 500–879 mg/dL [5.65–9.93 mmol/L]), and 10.2 for TG > 880 mg/dL [> 9.94 mmol/L] (vs. 9.9 for TG ≥ 880 mg/dL (≥ 9.94 mmol/L)) [5]. Accounting for differences in definitions employed for TG-specific subgroups, results from Patel et al. and Murphy et al. were largely similar as well [17, 18].

The present study, like those by Sanchez et al. and Patel et al., reported substantially higher rates of AP among patients with a history of this condition, and like Patel et al., rates were higher among younger versus older adults with sHTG. For example, among patients with a history of AP, the rate of AP (per 1,000 PYs) was 193 for the subgroup with TG ≥ 880 mg/dL (≥ 9.94 mmol/L) in the present study, 281 for the subgroup with TG > 880 mg/dL (> 9.94 mmol/L) in Sanchez et al., and 120 for the subgroup with TG 885–1770 (> 10–20 mmol/L) in Patel et al. We note that, irrespective of index TG value, a higher percentage of patients who experienced an AP event had baseline risk factors for AP (e.g., alcoholism, gallbladder disease, history of AP); they also had a higher prevalence of other comorbidities and were 1.5–2.5 times more likely to be on lipid-lowering medicine. AP risk was inversely associated with age (present study); AP rate was 17.0 among adults aged < 40 years with TG ≥ 880 mg/dL (≥ 9.94 mmol/L), versus 8.8 among adults aged ≥ 40 years with TG ≥ 880 mg/dL (≥ 9.94 mmol/L). AP rates were also found to be higher among adults with diabetes and those with a history of LLT.

While rates of CV events across TG-specific subgroups vary somewhat with previous research—presumably, due in large part to different algorithms for identifying CV events—trends are comparable [6, 7, 16, 18]. CVD rates were positively associated with TG levels, increasing from 3.3 to 10.3 per 1,000 PYs, although marginal increases in rates decreased with increasing TG levels (RR [vs. TG < 150 mg/dL (< 1.69 mmol/L)]: TG 150–499 mg/dL[1.69–5.64 mmol/L], 1.7; TG 500–879 mg/dL [5.65–9.93 mmol/L], 2.8; TG ≥ 880 mg/dL [≥ 9.94 mmol/L], 3.1). However, for adults aged < 40 years and those with a history of CV events, rates were markedly higher among patients with TG ≥ 880 mg/dL (≥ 9.94 mmol/L) (vs. TG 500–879 mg/dL [5.65–9.93 mmol/L]).

Several limitations of this study are noted. The study population was stratified into TG-specific subgroups based on a single TG value, for which fasting status was unknown. TG values were available for only a subset of patients in the MarketScan CCAE/MDCR Databases, and for those patients, TG values were available from only a subset of providers; moreover, lab results were limited to those obtained in the outpatient setting. However, a comparison of ambulatory claims with the lipid panel CPT code (CCAE/MDCR) versus tests for TG values (Labs) indicates that results for the majority (> 75%) of the tests conducted in the ambulatory setting are included in the study data source. In this study, attention was limited to AP/CV events requiring inpatient care (i.e., hospitalization) as prior research suggests that the positive predictive value of inpatient diagnoses is substantially higher than those rendered in an ambulatory setting [19–23]. Because AP/CV events requiring ambulatory care only (or those that were not medically attended in the outpatient/inpatient setting) were not considered, rates may be downwardly biased. Additionally, sample sizes for some subgroups were small, thus corresponding results should be interpreted in light of the wide confidence intervals. Adults with certain types of public health insurance (e.g., Medicaid, Medicare Advantage) and adults without health insurance are not represented in the study database; therefore, caution should be used when generalizing study results to other populations and settings.

## Conclusions

Rates of AP and CV events are substantially higher among adults with elevated TG values, and are especially high among adults with sHTG or eHTG, in particular those with these conditions who also have other risk factors (or proxies for risk factors). Understanding the magnitude of disease risk among sHTG/eHTG patients, with increasing levels of TGs as well as within important subgroups, is critical to improving patient care and outcomes.

## Supplementary Information

Supplementary Material 1.

## Data Availability

The study data sources are proprietary, provided by a third-party vendor via a license agreement with Avalere Health, and the authors do not have permission to disseminate the data without approval of the vendor. Inquiries regarding acquisition of the study data sources may be directed to the data vendor (Merative).
