# Supplementary material for: Rates of acute pancreatitis and cardiovascular events among adults with severe or extreme hypertriglyceridemia in US clinical practice
Source: Lipids Health Dis. 2025 Jul 28;24:252. doi: 10.1186/s12944-025-02658-8 (PMC12305886; doi:10.1186/s12944-025-02658-8)
Supplement: Supplementary file 1 — Supplementary Material 1. [file 12944_2025_2658_MOESM1_ESM.pdf]

Supplement -- Table 1. ICD-9/10 and CPT codes and operational algorithms for identifying AP and CV events

Condition	ICD-10 Codes	ICD-9 Codes	ICD-10-PCS	ICD-9-PCS	CPT	Algorithm
**AP**						Inclusion criteria: Principal diagnosis of AP or a principal diagnosis of an AP-related complication in combination with secondary diagnosis of AP in an acute-care setting
Acute pancreatitis	K85.0*-K85.3*, K85.8*, K85.9*	577.0	--	--	--	
AP-related complications					--	
Ascites	R18*	789.5*	--	--		
Acute necrotic colitis	K55.30	557.0	--	--	--	
Organ failure			--	--	--	
Heart failure	I11.0, I13.0, I13.2, I50*, I97.1	402.01, 402.11, 402.91, 428*, 429.4, 997.1	--	--	--	
Renal failure	N17*, N19*, N18.5, N18.6, I12.0, I13.11, Z99.2	584*, 585.5, 585.6, 586*	5A1D00Z, 5A1D60Z, 3E1M39Z	39.95, 54.98	90935-90940	
Respiratory failure	J96*	518.81, 518.83, 518.84	5A1935Z, 5A1945Z, 5A1955Z	96.70-96.72	94002-94005	
Other organ failure	K72*, K70.40, K40.41 K90.82*, K90.83, G93.89	--	--	--	--	
Sepsis	A41*, R65.2*, A02.1, A20.7, A22.7, A26.7, A32.7, A39.4, A40*, A42.7, A54.86, B37.7, R78.81, T81.12*, T81.44*	038*, 995.91, 995.92, 785.52, 020.2, 022.3, 036.2, 790.7, 998.02	--	--	--	
Systemic inflammatory response syndrome (SIRS)	A40, A41, R65, T81.12	995.9, 998.02, 038, 785.52	--	--	--	
Other	F10.129, F10.231, R10.9	305.00, 291.0, 789.00	--	--	--	
**CV events**						Inclusion criteria: Principal diagnosis code for heart disease (e.g., myocardial infarction, unstable angina), cerebrovascular disease (e.g., stroke, transient ischemic attack [TIA]), or heart failure
Peripheral vascular disease	I73.9	443.9	--	--	--	
Heart failure	I11.0, I50*, I97.1	402.01, 402.11, 402.91, 428*, 429.4, 997.1	--	--	--	
Acute myocardial infarction	I21*, I22*	410*	--	--	--	
Stroke	I63*	433.01, 433.11, 433.21, 433.31, 433.81, 433.91, 434.01, 434.11, 434.91	--	--	--	
TIA	G45.9	435.9	--	--	--	
Unstable angina	I20.0	411.1	--	--	--	
Acute coronary syndrome/ischemic heart dis[illegible]	I21*-124*	410*, 411*, 429.5-429.7*	--	--	--	

Supplement -- Table 2. ICD-10 and ICD-9 Codes for Comorbid Conditions

Condition	ICD-10 Codes	ICD-9 Codes
Alcoholism	F10.11*-F10.12*, F10.14-F10.92*, F10.94-F10.99, G62.1, I42.6, K29.2*, K70*, R78.0	291*, 303*, 305.0*, 357.5, 425.5, 535.3*, 571.0-571.3, 790.3, E860.0, E860.1
Atherosclerosis	I25.708, I25.709, I25.710, I25.711, I25.718, I25.719, I25.720, I25.721, I25.728, I25.729, I25.730, I25.731, I25.738, I25.739, I25.750, I25.751, I25.758, I25.759, I25.760, I25.761, I25.768, I25.769, I25.790, I25.791, I25.798, I25.799, I25.81*-I25.84, I67.82	440.22, 414.0*, 414.3, 429.2, 437.0, 440*, 707.11-707.19, 707.8, 785.4
Cardiovascular disease		
Cerebrovascular disease	G45*, G97.3*, I60*-I66*, I67.2, I67.8*-I67.9, I69*, I73.9, I97.81*-I97.82* (Excl. I67.83)	430-438 (Excl. 437.2-437.6)
Heart disease (chronic)	I05*-I09*, I11*, I13*, I20*-I25*, I27*, I34-I39*, I42*-I43, I50*, I51.0, I51.3, Q20*-Q24*, Q25.1, Q25.21	393-398*, 402*, 404*, 410*-414*, 416*, 421.1, 424*-425*, 428*, 429.2, 429.5- 429.7*, 429.89, 745*-746*, 747.11
Gallbladder disease	K80*-K83	574-576
Hyperthyroidism	E05.90, E05.91	242.90, 242.91
Hypothyroidism	E89.0, E03.2, E03.8, E03.9	244.0-244.9
Immunosuppressive conditions/treatments		
Neoplasms	C00* - C96* (Excl. C44*, C88.0, C96.5, C96.6)	140*-208* (Excl. 173)
Encounter for radiation/chemotherapy/immunotherapy	Z51.0-Z51.1*	V58.0-V58.1*
Transplanted organ and tissue status	Z94* (Excl. Z94.5, Z94.6, Z94.7)	V42.0-V42.1, V42.6-V42.9*
Liver disease (chronic)		
NASH/NAFLD	K75.81, K76.0	571.8, 573.3
All others	I85*, K70*, K72* - K74*, K75.0-K75.1, K75.4, K75.9, K76.1, K76.6-K76.7, K76.89, K76.9	456.0-456.2*, 570-572*, 573.0, 573.8, 573.9 (Excl. 571.8)
Malabsorption	K90*	579*
Metabolic disorders		
Diabetes (exclude pregnancy)	E08* - E13*, Z79.4	249*-250*, V58.67
Disorders of lipoprotein metabolism and other lipidemias	E78*	272.0-272.5, 272.8-272.9, 759.89
Hypertensive diseases	I10 - I16*	401.*-405.*
Hyperglycemia, unspecified	R73.9	790.29
Overweight or obese	E66*, O99.21*, Z68.3*, Z68.4*	278.0*, 649.1*, V85.3*, V85.4*
Neurologic disorders (chronic)	F01*-F09, G12*-G14, G20-G23*, G30*-G32.*, G35-G37.*, G40*, G80, I63*, I66*, Q00*, Q05.0-Q05.8	138, 290*, 293*-294*, 310.0-310.2, 310.89-310.9, 323.9, 330.8-330.9, 331.0-331.2, 331.6-331.7, 331.82-333.0, 333.71, 333.92, 334.4, 335*, 336.2, 340-341*, 343*, 345*, 349.89, 357.3, 438*, 740*
Osteoarthritis	M15*-M19*	715*
Pancreatitis		
Acute	K85*	577.0
Chronic	K86.0, K86.1	577.1
Renal disease (chronic)	E10.2*, E11.2*, E13.2*, I12.9, I13*, K76.7, N03*-N08, N14*-N19, N25*, Q60*, R80*, Z49*, Z91.15, Z94.0, Z99.2 (Excl. N15.1, Q60.6, R80.2)	249.40, 250.4*, 403.00, 403.10, 403.90, 404*, 581*-588*, 590.81, 590.9, 753.0, 791.0, V42.0, V45.1*, V56*
Respiratory disease	E84*, I27.2*, I27.81, I27.89, I27.9, J40 - J47*, J60 - J66*, J68.4, J84*, J96.1*-J96.2*, P27	277.0*, 416.8-416.9, 490-494*, 496, 500-505, 506.4, 515-516*, 518.53, 518.83-518.84, 770.7

Supplement -- Table 3. Rates and relative rates of AP and CV events by index TG value, overall and for those with additional risk factors

	Index TG Value (mg/dL)			
	<150 mg/dL (<1.69 mmol/L)	150-499 mg/dL (1.69-5.64 mmol/L)	500-879 mg/dL (5.65-9.93 mmol/L)	≥880 mg/dL (≥9.94 mmol/L)
**AP***				
Overall	(N=1,290,630)	(N=449,119)	(N=12,050)	(N=3,944)
Rate (95% CI) per 1,000 person-years	0.6 (0.5-0.6)	1.1 (1.0-1.1)	3.1 (2.5-4.0)	9.9 (7.6-12.9)
Relative rate (95% CI)	--	1.9 (1.7-2.0)	5.5 (4.3-7.1)	17.4 (13.3-22.8)
Age <40 Years	(N=372,749)	(N=84,353)	(N=2,258)	(N=853)
Rate (95% CI) per 1,000 person-years	0.3 (0.2-0.3)	0.9 (0.7-1.1)	6.3 (3.7-10.6)	17.0 (10.9-26.5)
Relative rate (95% CI)	--	3.2 (2.5-4.1)	21.9 (12.7-37.6)	59.3 (37.2-94.7)
Age ≥40 Years	(N=917,881)	(N=364,766)	(N=9,792)	(N=3,091)
Rate (95% CI) per 1,000 person-years	0.7 (0.6-0.7)	1.1 (1.0-1.2)	2.5 (2.0-3.3)	8.8 (6.3-12.3)
Relative rate (95% CI)	--	1.6 (1.5-1.79)	3.8 (2.9-4.99)	13.3 (9.5-18.59)
Diabetes Hx	(N=166,342)	(N=101,573)	(N=4,231)	(N=1,541)
Rate (95% CI) per 1,000 person-years	1.1 (1.0-1.2)	1.7 (1.5-1.9)	4.5 (3.2-6.2)	13.9 (10.2-19.0)
Relative rate (95% CI)	--	1.5 (1.3-1.7)	4.0 (2.8-5.6)	12.2 (8.8-17.0)
AP Hx	(N=2,464)	(N=1,390)	(N=97)	(N=86)
Rate (95% CI) per 1,000 person-years	42.6 (35.8-50.8)	61.1 (50.1-74.5)	137.8 (84.5-224.5)	193.0 (127.3-292.8)
Relative rate (95% CI)	--	1.4 (1.1-1.9)	3.2 (1.9-5.4)	4.5 (2.9-7.1)
LLT Hx	(N=208,442)	(N=121,481)	(N=3,942)	(N=1,304)
Rate (95% CI) per 1,000 person-years	0.8 (0.7-0.9)	1.4 (1.2-1.5)	5.3 (3.8-7.4)	13.9 (9.4-20.6)
Relative rate (95% CI)	--	1.70(1.47-1.97)	6.59(4.65-9.33)	17.19(11.45-25.82)
**CV****				
Overall	(N=1,290,630)	(N=449,119)	(N=12,050)	(N=3,944)
Rate (95% CI) per 1,000 person-years	3.3 (3.3-3.4)	5.8 (5.6-5.9)	9.5 (8.3-10.8)	10.3 (8.3-12.8)
Relative rate (95% CI)	--	1.7 (1.7-1.8)	2.8 (2.5-3.2)	3.1 (2.5-3.8)
Age <40 Years	(N=372,749)	(N=84,353)	(N=2,258)	(N=853)
Rate (95% CI) per 1,000 person-years	0.3 (0.3-0.4)	0.8 (0.7-1.0)	1.6 (0.8-3.1)	3.0 (1.2-7.1)
Relative rate (95% CI)	--	2.5 (2.0-3.1)	4.8 (2.4-9.6)	9.0 (3.7-21.8)
Age ≥40 Years	(N=917,881)	(N=364,766)	(N=9,792)	(N=3,091)
Rate (95% CI) per 1,000 person-years	4.3 (4.2-4.4)	6.7 (6.5-6.9)	11.0 (9.6-12.5)	11.6 (9.3-14.5)
Relative rate (95% CI)	--	1.5 (1.5-1.6)	2.5 (2.2-2.9)	2.7 (2.1-3.3)
Diabetes Hx	(N=166,342)	(N=101,573)	(N=4,231)	(N=1,541)
Rate (95% CI) per 1,000 person-years	10.0 (9.6-10.3)	13.0 (12.5-13.5)	17.7 (15.1-20.7)	18.1 (14.0-23.4)
Relative rate (95% CI)	--	1.3 (1.2-1.4)	1.8 (1.5-2.1)	1.8 (1.4-2.4)
CV Hx	(N=5,987)	(N=2,482)	(N=65)	(N=27)
Rate (95% CI) per 1,000 person-years	65.9 (60.8-71.4)	76.3 (68.1-85.4)	85.9 (45.0-163.7)	116.5 (61.6-220.5)
Relative rate (95% CI)	--	1.2 (1.0-1.3)	1.3 (0.7-2.5)	1.8 (0.9-3.4)
LLT Hx	(N=208,442)	(N=121,481)	(N=3,942)	(N=1,304)
Rate (95% CI) per 1,000 person-years	8.1 (7.8-8.3)	9.0 (8.7-9.4)	13.0 (10.8-15.7)	14.5 (10.6-19.7)
Relative rate (95% CI)	--	1.1 (1.1-1.2)	1.6 (1.3-2.0)	1.8 (1.3-2.4)

AP: acute pancreatitis; CI: confidence interval; CV: cardiovascular event; Hx: history; LLT: lipid-lowering therapy; TG: trigylceride

*Events with inpatient principal AP diagnosis or inpatient principal AP-complication diagnosis and secondary AP diagnosis

**Events with inpatient principal diagnosis

Supplement -- **Table 4.** Rates and relative rates of AP and CV events by index TG value based on alternative stratification scheme

	Index TG Value (mg/dL)				
	<150 mg/dL (<1.69 mmol/L)	150-499 mg/dL (1.69-5.64 mmol/L)	500-879 mg/dL (5.65-9.93 mmol/L)	880-1771 mg/dL (9.94-19.99 mmol/L)	>1771 mg/dL (>20 mmol/L)
**AP***					
Overall	(N=1,290,630)	(N=449,119)	(N=12,050)	(N=2,661)	(N=1,283)
Rate (95% CI) per 1,000 person-years	0.6 (0.5-0.6)	1.1 (1.0-1.1)	3.1 (2.5-4.0)	9.1 (6.5-12.6)	12.0 (7.8-18.5)
Relative rate (95% CI)	--	1.9 (1.7-2.0)	5.5 (4.3-7.1)	15.9 (11.4-22.3)	21.1 (13.6-32.6)
**CV****					
Overall	(N=1,290,630)	(N=449,119)	(N=12,050)	(N=2,661)	(N=1,283)
Rate (95% CI) per 1,000 person-years	3.3 (3.3-3.4)	5.8 (5.6-5.9)	9.5 (8.3-10.8)	10.2 (7.8-13.3)	10.6 (7.4-15.2)
Relative rate (95% CI)	--	1.7 (1.7-1.8)	2.8 (2.5-3.2)	3.1 (2.4-4.0)	3.2 (2.2-4.6)

AP: acute pancreatitis; CI: confidence interval; CV: cardiovascular event; TG: trigylceride

*Events with inpatient principal AP diagnosis or inpatient principal AP-complication diagnosis and secondary AP diagnosis

**Events with inpatient principal diagnosis
